# Protocol for measuring endogenous 24(S),25-epoxycholesterol and inducing innate immune memory in mouse macrophages

**DOI:** 10.1016/j.xpro.2025.104024

**Published:** 2025-08-09

**Authors:** Yongxiang Liu, Huan Jin, Bitao Huo, Xiaojun Xia, Jinyun Liu

**Affiliations:** 1 State Key Laboratory of Oncology in South China, Guangdong Provincial Clinical Research Center for Cancer, Sun Yat-sen University Cancer Center, Guangzhou, P.R. China; 2 Guangzhou National Laboratory, Guangzhou International Bio-Island, Guangzhou, P.R. China; 3 Hainan Academy of Medical Sciences, Hainan Medical University, Haikou, P.R. China

**Keywords:** Cell Biology, Immunology, Metabolism, model Organisms, Molecular Biology

## Abstract

Multiple metabolic pathways and metabolites are involved in innate immune memory induction of macrophages; however, protocols for *in vitro*-trained immunity assays induced by metabolites in mouse macrophages are limited. Here, we present a protocol for measuring endogenous 24(S),25-epoxycholesterol and inducing innate immune memory in mouse macrophages. We describe steps for sample preparation, measurement of 24(S),25-epoxycholesterol, and establishment of an *in vitro*-trained immunity model. We then detail procedures for assays measuring cytokine concentration and for assay for transposase-accessible chromatin using sequencing (ATAC-seq).

For complete details on the use and execution of this protocol, please refer to Liu et al.^1^

## Highlights

• Instructions on culturing and differentiation of bone marrow-derived macrophages (BMDMs)
• Workflow for precise quantification of endogenous 24(S),25-EC using LC-MS
• Validation procedures for establishing metabolite-induced trained immunity in BMDMs

## Abstract (editor highlights)

Publisher’s note: Undertaking any experimental protocol requires adherence to local institutional guidelines for laboratory safety and ethics.

## Graphical abstract

**Figure:**
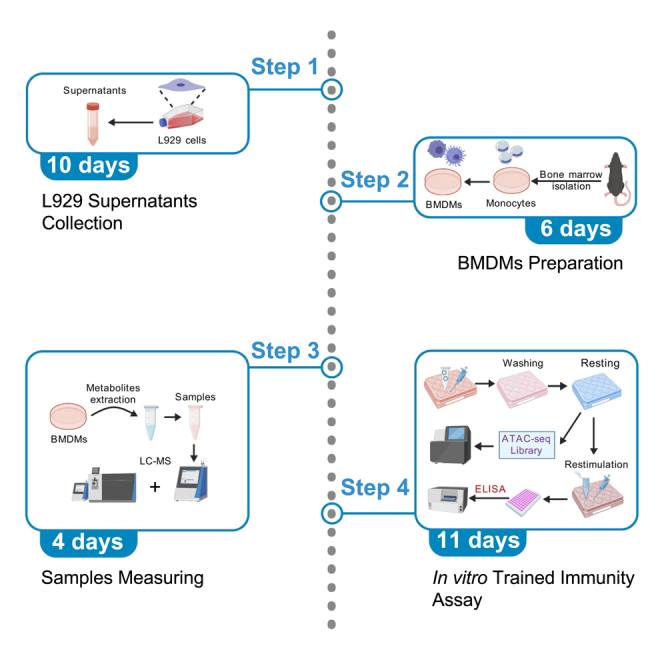

## Before you begin

Innate immune memory refers to the memory status that is ready for responding to the encountering specific or irrelevant secondary stimuli,^2^ and this state is primarily characterized by epigenetic reprogramming.^3^ In this protocol, we take advantage of assay for transposase accessible chromatin using sequencing (ATAC-seq) to examine chromatin accessibility and to characterize innate immune memory with enhanced openness.^4^ Whole glucan particles (WGP) are a well-established inducer of trained immunity^1^^,^^5^ and serve as a positive control here for ATAC-seq assay. We conducted all the assays using mouse bone marrow-derived macrophages (BMDMs) and the experiments were approved by Institutional Animal Care and Use Committee (IACUC) of Sun Yat-sen University.

### Institutional permissions

The procedures of the experiments were approved by Institutional Animal Care and Use Committee (IACUC) of Sun Yat-sen University.

**Figure 1:**
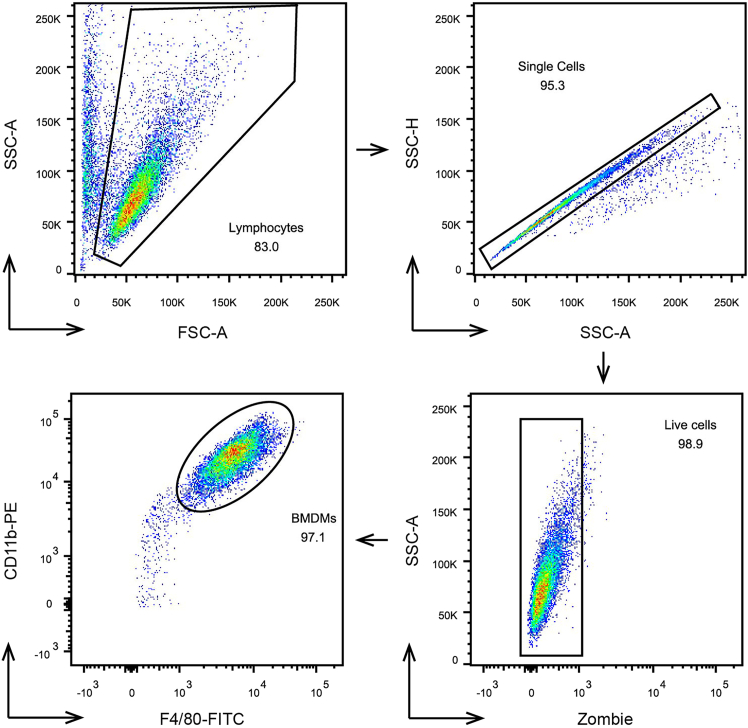
Gating strategy of *in vitro* differentiated BMDMs (Zombie^−^ F4/80^+^ CD11b^+^)

### Culturing of L929 cells and supernatant collection

**Timing: about 10 days**

1. Supplement the complete DMEM medium with GlutaMax to create DMEM-G medium.
2. Thaw the cryopreserved L929 cells quickly in a warming bath at 37°C.
3. Transfer the cells to a 15 mL tube and add complete DMEM medium to a total volume of 5 mL.
4. Centrifuge the tube at 300 × *g* for 3 min at 25°C.
5. Remove the supernatants and resuspend the cell pellets with complete DMEM medium, then transfer them to a T25 flask with a total volume of 5 mL medium.
6. Culture the cells in a 37°C incubator with 5% CO_2_ for 3 days. Replace the medium every 3 days until the cells are fully confluent.
7. Digest the cells with 0.25% Trypsin-EDTA (1 mL for a T25 flask) in a 37°C incubator for 1 min, then quench the digestion with 4 mL of complete DMEM.
8. Transfer the cells to a 15 mL tube and centrifuge cells as in Steps 3 and 4, and resuspend the cell pellets with 1 mL of complete DMEM.
9. Count the cells using the TC20 Automated Cell Counter (Bio-Rad) following the manufacturer’s instruction.
10. Adjust 1 × 10^5^ cells to a T75 flask with total volume of 15 ml of DMEM-G medium and cultured for 7 days in a 37°C incubator with 5% CO_2_.
11. Collect and filter the supernatants using a 0.45 μm syringe filter to obtain L929 conditional medium which contains M-CSF.

**CRITICAL:** To obtain L929 conditional medium with sufficient M-CSF, the cells are cultured for 7 days without replacing or adding fresh medium. Supernatants must be filtered for collection to avoid contamination by L929 cells.

***Note:*** The supernatants can be used immediately or stored at −80°C for up to 6 months, but avoid freeze-thaw cycle.

### Isolation and differentiation of BMDMs *in vitro*

**Timing: about 6 days**

12. Prepare the culture medium for BMDMs (referred to as BMDM-CM), ACK (ammonium-chloride-potassium) lysis buffer for erythrocytes removal and FACS (fluorescence activated cell sorting) staining buffer.
13. Euthanize C57BL/6J (B6) mice using CO_2_ and subsequently collect femur and tibia from hinder legs with tissues and skins peeled off.
14. Submerge the bare femur and tibia in 75% alcohol for 1–2 min and rinse with 1 × PBS twice.
15. Cut the femur and tibia into two fragments at the site of arthrosis.
16. Use a 1 mL syringe equipped with a 25 G needle to flush out cells from marrow with 1 × PBS into 100 mm cell culture dishes. Repeat this process with each side for 5 times.
17. Disperse the cell cluster by pipetting up and down using a 1 mL pipette for 10 times.
18. Filter the cells into a 50 mL tube with a 70 μm cell strainer, and then centrifuge the tube with 300 × *g* for 5 min at 25°C.
19. Discard the supernatants and resuspend the cells with 1 mL of ACK lysis buffer for 1 min.
20. Add 5 mL of PBS to quench the lysis, followed by centrifugation (300 × *g*, 5 min, 25°C) again.
21. Resuspend the cell pellets in 25 mL of BMDM-CM, and culture the cells in two 6-well plates at a 37°C incubator with 5% CO_2_ for differentiation.
22. Add an additional 2 mL of BMDM-CM on the third day and collect the differentiated BMDMs on the fifth day.
23. Digest the BMDMs with 0.25% Trypsin-EDTA (300 μL per well) in a 37°C incubator for 5 min, then quench the digestion with 1 mL of BMDM-CM, followed by centrifugation (300 × *g*, 5 min, 25°C).
24. Resuspend the cell pellets with 1 mL FACS staining buffer and filter the cells through a 100 μm cell strainer to gain single cell suspension.
25. Block the Fc receptor (FcR) with mouse anti-CD16/32 antibody (5 μg/mL) at 25°C for 10 min and then wash the cells with FACS staining buffer twice by centrifugation (300 × *g*, 5 min, 25°C).
26. Stain the cells with Zombie NIR dye and two fluorescence-labeled antibodies against F4/80 (FITC, 5 μg/mL) and CD11b (PE, 5 μg/mL) in FACS staining buffer at 4°C for 30 min in the dark.
27. Wash the cells twice as pre Step 23, then resuspend the cells in FACS staining buffer and subject to flow cytometry analysis using BD LSR Fortessa X-20 Cell Analyzer.
28. Analyze the fluorescence data using the FlowJo software X10.0.7r2 (Tree Star), with an example showing that the differentiated BMDMs constitute over 97% of total cells (Figure 1).

## Key resources table

**Table:** 

REAGENT or RESOURCE	SOURCE	IDENTIFIER
**Antibodies**		

Anti-mouse CD11b-PE (M1/70; use 5 μg/mL)	BioLegend	Cat#101208; RRID:AB_312791
Anti-mouse F4/80-FITC (BM8; use 5 μg/mL)	BioLegend	Cat#123108; RRID:AB_893502
Anti-mouse CD16/32 (93; use 5 μg/mL)	BioLegend	Cat#101301; RRID:AB_312800

**Chemicals, peptides, and recombinant proteins**		

DMEM medium	Gibco	Cat#11965092
RPMI 1640 medium	Gibco	Cat#11875093
L929 conditional medium	this protocol	N/A
Ammonium chloride (NH_4_Cl)	Sigma-Aldrich	Cat#213330
Potassium bicarbonate (KHCO_3_)	Sigma-Aldrich	Cat#237205
Sodium chloride (NaCl)	Sigma-Aldrich	Cat#S9888
EDTA (0.5 M)	Sangon Biotech	Cat#B540625
1 × PBS	Gibco	Cat#10010023
Fetal bovine serum (FBS)	Gibco	Cat#10270106
0.25% Trypsin-EDTA	Gibco	Cat#25200072
Penicillin-Streptomycin	Gibco	Cat#15070063
β-mercaptoethanol	Gibco	Cat#21985023
Sodium pyruvate	Gibco	Cat#11360070
GlutaMAX	Gibco	Cat#35050061
Antibiotic-Antimycotic	Gibco	Cat#15240062
Lipopolysaccharides (LPS)	Sigma-Aldrich	Cat#L2630
Pam3CSK4	InvivoGen	Cat#tlrl-pms
IFN-γ	PeproTech	Cat#315-05
Hexane	Fisher Scientific	Cat#60-010-54
Isopropanol	Fisher Scientific	Cat#A461-500
Methanol	Fisher Scientific	Cat#A452-4
Formic acid	Fisher Scientific	Cat#A117-2AMP
Ethanol	Sangon Biotech	Cat#A500737
24(S),25-epoxycholesterol	Abcam	Cat#ab141633
WGP dispersible	InvivoGen	Cat#tlrl-wgp
TRIzol	Invitrogen	Cat#15596026
Novoprotein Tagment DNA Extract Beads	Novoprotein	Cat#N245
Novoprotein DNA Clean Beads	Novoprotein	Cat#N240

**Critical commercial assays**		

TNF-α Mouse Uncoated ELISA Kit	Invitrogen	Cat#88-7324-88
IFN-γ Mouse Uncoated ELISA Kit	Invitrogen	Cat#88-7314-88
Mouse IFN-β bioluminescent ELISA kit 2.0	InvivoGen	Cat#luex-mifnbv2
Chromatin Profile Kit for Illumina	Novoprotein	Cat#N248
Novoprotein NovoNGS Index kit for Illumina	Novoprotein	Cat#N239
Zombie NIR Fixable Viability Kit	BioLegend	Cat#423105
Cell Counting Kit-8	GOONIEBIO	Cat#100-120

**Experimental models: Cell lines**		

L929 cell line	a gift from Prof. Jun Chen (Sun Yat-sen University Zhongshan School of Medicine)	N/A

**Experimental models: Organisms/strains**		

Mouse: C57BL/6J (6–8 weeks; female or male)	Vital River Laboratory	N/A

**Software and algorithms**		

GraphPad Prism version 9.5.1	GraphPad Software	https://www.graphpad.com/features
FlowJo software X10.0.7r2	Tree Star	https://www.flowjo.com/flowjo/download
MassHunter Quantitative Analysis Software	Agilent	https://www.agilent.com/en/product/software-informatics/mass-spectrometry-software/data-analysis/quantitative-analysis
Generic Diagramming Platform^6^	BioGDP	https://biogdp.com/

## Materials and equipment

**Table:** DMEM-G

Reagent	Final concentration	Amount
DMEM medium	N/A	444.5 mL
FBS	10%	50 mL
Penicillin-Streptomycin	1%	5 mL
GlutaMax	2 mM	0.5 mL
Total	N/A	500 mL

Store at 4°C for up to 4 weeks.

**Table:** BMDM-CM

Reagent	Final concentration	Amount
RPMI 1640 medium	N/A	388.5 mL
FBS	10%	50 mL
Penicillin-Streptomycin	1%	5 mL
L929 conditional medium	10%	50 mL
Antibiotic-Antimycotic	1%	5 mL
β-Mercaptoethanol	55 μM	0.5 mL
Sodium Pyruvate	1 mM	0.5 mL
GlutaMax	2 mM	0.5 mL
Total	N/A	500 mL

Store at 4°C for up to 4 weeks.

***Alternative:*** The final concentration of L929 conditional medium can be adjusted from 10 to 20%. Additionally, L929 conditional medium can be substituted with M-CSF at a final concentration of 20–40 ng/mL.

**Table:** ACK lysis buffer

Reagent	Final concentration	Amount
NH_4_Cl	150 mM	8.02 g
KHCO_3_	10 mM	1 g
EDTA (0.5 M)	0.1 mM	200 μL
Total	N/A	1 L

Store at 25°C–30°C for 1 year.

***Note:*** Sterilize using a 0.22 μm syringe filter before use.

***Alternative:*** The powder of EDTA or Na_2_EDTA can also be dissolved for ACK, but the final pH should be adjusted between 7.2 and 7.4.

**Table:** FACS staining buffer

Reagent	Final concentration	Amount
1 × PBS (sterilized)	N/A	48.8 mL
FBS	2%	1 mL
EDTA (0.5 M)	2 mM	200 μL
Total	N/A	50 mL

Store at 4°C for up to 2 weeks.

***Alternative:*** 0.5% BSA can serve as alternative for 2% FBS.

**Table:** ATAC lysis buffer

Reagent	Final concentration	Amount
25 × Lysis buffer	1 ×	2 μL
10% NP-40	0.1%	0.5 μL
10% Tween-20	0.1%	0.5 μL
1% Digitonin	0.01%	0.5 μL
Sterilized ddH_2_O	N/A	46.5 μL
Total	N/A	50 μL

Keep on ice after fresh reconstitution and use immediately.

**Table:** ATAC washing buffer

Reagent	Final concentration	Amount
25 × lysis buffer	1 ×	40 μL
10% NP-40	0.1%	10 μL
Sterilized ddH_2_O	N/A	950 μL
Total	N/A	1 mL

Keep on ice after fresh reconstitution and use immediately.

**Table:** ATAC tagmentation buffer

Reagent	Final concentration	Amount
3 × PBS	0.3 ×	5 μL
10% Tween 20	0.1%	0.5 μL
1% Digitonin	0.01%	0.5 μL
Sterilized ddH_2_O	N/A	17 μL
2 × TD buffer	1 ×	25 μL
Tn5 Transposome Mix	N/A	2 μL
Total	N/A	50 μL

Keep on ice after fresh reconstitution and use immediately.

***Note:*** The above three buffers included in the Chromatin Profile Kit for Illumina (Cat#N248, Novoprotein) should be freshly reconstituted and gently mixed with a pipette to avoid foam formation.

***Alternative:*** Buffers vary among different commercial kits. Furthermore, Hyperactive ATAC-Seq Library Prep Kit for Illumina (Cat#TD711-01, Vazyme) and ATAC-Seq Kit (Cat#53150, Active Motif) are also recommended for use in ATAC-seq assays.

## Step-by-step method details

### Sample preparation for measuring endogenous 24(S),25-epoxycholesterol

**Timing: about 3 days**

This step outlines the procedure for preparing samples for mass spectrometric (MS) measurement. Consequently, the hexane and isopropanol used should be of LC-MS grade, and the 2 mL EP tube should be of low binding quality. Prior to cell harvesting, ensure that the hexane and isopropanol are pre-cooled to −20°C and kept over 8 h.

1. Digest the mature BMDMs following the previous steps and count the cells using the TC20 Automated Cell Counter (Bio-Rad) according to the manufacturer’s instruction.
2. Adjust 1 × 10^7^ cells to a 100 mm dish and culture the cells in a 37°C incubator with 5% CO_2_. Each group requires at least 5 replicates.
3. After resting for 24 h, the BMDMs are either harvested directly or treated for the indicated durations.
4. Mix the pre-cooled hexane (0.6 volume) and isopropanol (0.4 volume) thoroughly and place the mixture on ice prior to use.
5. Remove the medium from the dish and wash the BMDMs twice with pre-chilled 0.9% saline.

***Note:*** Sodium chloride is dissolved in ultra-pure H_2_O and sterilized using a 0.22 μm syringe filter. Store at 4°C prior to use.

6. Add 1 mL of the hexane/isopropanol mixture to the dish and quickly scrape the cells with a cell scraper on ice, then transfer the contents to a new 2 mL EP tube.

**CRITICAL:** Since the organic mixture is prone to volatilization, this process must be conducted swiftly, with a duration of less than 30 s being ideal. To prevent operational errors, the routes and times for scraping each dish should be kept similar.

7. Vortex the scraped mixture at the highest speed for 3 min and chill the EP tube on ice for 2 min.
8. Centrifuge the samples at 15,000 × *g* for 10 min at 4°C.
9. Delicately draw the organic supernatants into a new EP tube and then place it on ice.

***Note:*** After centrifugation, the mixture separates, carefully transfer the supernatants to a new tube. To avoid contamination by sediments, it is advised to leave approximately 50 μL of supernatant residue.

10. Open the tube and dry the mixture thoroughly by blowing nitrogen gas using Reacti-Vap Evaporators (Thermo Scientific).

***Note:*** The dried sample is subjected to following measurement immediately or can be stored at −80°C for up to one week.

### Sample measurement of 24(S),25-epoxycholesterol

**Timing: about 1 day**

This method is customized for measuring 24(S),25-epoxycholesterol (24(S),25-EC) in the organic mixture. For other lipid metabolites, the procedures and conditions should be explored case by case accordingly.

**Figure 2:**
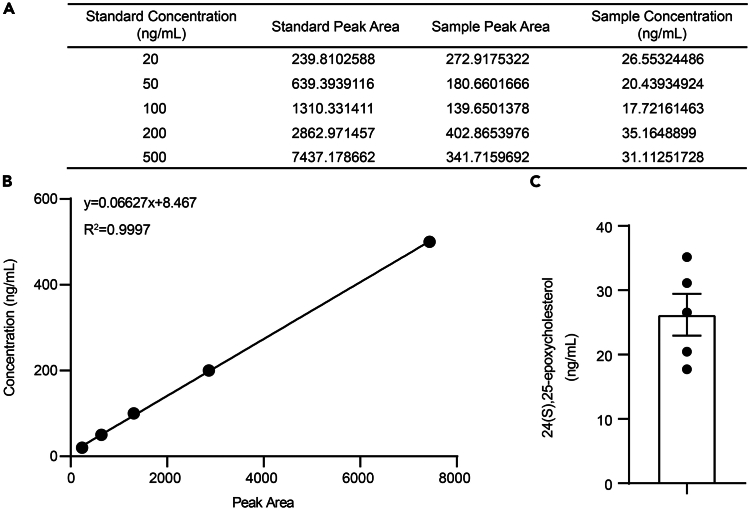
Measurement of endogenous 24(S),25-EC in BMDMs (A) The peak area of gradient-diluted standards and samples. (B) The standard curve for the measured 24(S),25-EC standards. (C) Endogenous level of 24(S),25-EC in BMDMs. Mean ± SEM with five independent samples.

11. Reconstitute the dried samples with 100 μL of LC-MS grade methanol and vortex thoroughly.
12. Utilize the Agilent 6495A Triple Quadrupole mass spectrometer, coupled with an Agilent 1290 Infinity II high-performance liquid chromatography (HPLC) system with an Agilent C18 column (2.1 × 50 mm, 3.0 μm), for the measurement of 24(S),25-EC.
  a. Upload 1 μL of the reconstituted sample or gradient-diluted standard of commercial 24(S),25-EC (Figure 2A) into an Agilent C18 column and keep the temperature of column at 30°C.
  b. The gradient program and flow rate are shown below:

**Table:** 

Time (min)	Flow rate (mL/min)	Percentage in phase A	Percentage in phase B
0.00	0.40	15	85
10.00	0.40	0	100
12.90	0.40	0	100
13.00	0.40	15	85
Post-time	0.40	2 min

    ***Note:*** Phase A comprises 90% (v/v) ultra-pure water and 10% methanol (including 0.05% formic acid), while phase B is composed of methanol with 0.05% formic acid.
  c. Quantify the samples using multi reaction monitoring (MRM) mode in positive mode via atmospheric pressure chemical ionization (APCI).
    ***Note:*** The MS parameters are set as follows: a capillary voltage of 3,000 V, a gas temperature of 250°C, a gas flow rate of 11 L/min, a vaporizer temperature of 450°C, and a nebulizer pressure of 35 psi.
  d. Acquire the peak area by collecting ions with a precursor ion set to 383.3 m/z, a product ion of 95 m/z, and a collision energy of 40 eV.
  e. Process the data using Agilent MassHunter Quantitative Analysis software and calculate the concentration by peak area, based on the standard curve from serially diluted standards (Figure 2).
    **CRITICAL:** To obtain reliable results, the value of the peak area should fall within the range of the standard curve, thus warranting an appropriate pre-test dilution for the samples.
    ***Alternative:*** A negative control, such as samples pre-treated by inhibitors of enzymes involved in synthesizing the metabolites to be measured, is suggested for the assay.

**Figure 3:**
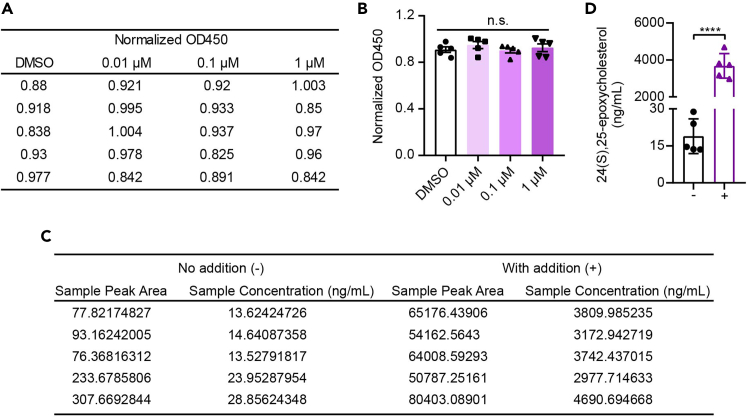
Measurement of intracellular 24(S),25-EC with addition of exogenous 24(S),25-EC (A) The measured values of the CCK-8 assay in BMDMs treated with various concentrations of 24(S),25-EC for 24 h. (B) The normalized OD450 of the CCK-8 reaction assay in BMDMs. Mean ± SEM with five independent samples, by one-way ANOVA test followed by Tukey’s multiple comparisons test. n.s., not significant. (C) The peak area of samples with or without the addition of 24(S),25-EC. (D) The intracellular level of 24(S),25-EC in BMDMs. Mean ± SEM with five independent samples, by unpaired t test. ∗∗∗∗p < 0.0001.

### Establishing an *in vitro*-trained immunity model using 24(S),25-epoxycholesterol

**Timing: about 8 days**

**CRITICAL:** To investigate the role of endogenous metabolites in inducing innate immune memory, it is essential to measure whether these metabolites can enter cells when directly added to medium. If not, permeable derivatives of the metabolites are recommended for further exploration.

13. Add 24(S),25-EC (1 μM) to the BMDMs in culture medium directly and incubate for 24 h.

**CRITICAL:** Before conducting the assay, it is recommended to perform CCK-8 assay to evaluate the impact of metabolites on cellular activity and verify that the concentration is optimal. Besides, freshly generated BMDMs are used to ensure the reliability of the assay.

***Note:*** Prepare fresh BMDMs at an appropriate density the day before this assay. For measuring 24(S),25-EC, a 10 cm dish seeded with 1 × 10^7^ cells is recommended. For trained immunity assay, a 24-well culture plate with 5 × 10^5^ cells per well is suggested.

14. Harvest the cells from 10 cm dishes and subject them for 24(S),25-EC measurement using the aforementioned procedures (Figure 3).
15. Wash the cells in the 24-well plate twice with pre-warmed 1 × PBS.
16. Culture the washed cells for another 7 days (resting period) with BMDM-CM at a 37°C incubator with 5% CO_2._ Replace half of the medium with fresh BMDM-CM every 3 days.
17. After resting, wash the cells once with 1 × PBS and add fresh medium containing LPS (100 ng/mL).

***Alternative:*** Non-specific stimuli are often used for secondary stimulation. LPS, a TLR4 agonist or Pam3CSK4, a TLR2 agonist, is commonly used. IFN-γ priming prior to restimulation is selective^7^ and a rest period of 5–7 days is acceptable.

18. Collect the culture supernatants after 24 h for ELISA assays immediately or freeze the samples at −20°C.

***Note:*** For RNA isolation, the cells are harvested with TRIzol reagent at 4–6 h post restimulation. The RNA is used for either qPCR analysis or transcriptomic sequencing.

### ELISA assay measuring cytokine concentration

**Timing: about 2 days**

The ELISA tests are performed in accordance with the guidelines provided by the manufacturer for the commercial kits listed in the key resources table.

19. Coat 100 μL of diluted capture antibodies into high-binding 96-well plates with 1 × coating buffer and incubate over 16 h at 4°C.

***Alternative:*** 1 × coating buffer can be substituted with 1 × PBS (pH 7.4) buffer. Both buffers should be filtered through a 0.22 μm syringe filter.

**CRITICAL:** Coating buffer must be free of any carrier proteins, including BSA and FBS. Besides, high-binding plates are crucial for the effective binding of antibodies.

20. Wash the plates 3 times with 200 μL washing buffer per well and tap the plates on absorbent paper towels to remove any residual buffer.

***Note:*** The washing buffer is composed of 1 × PBS with 0.05% (v/v) Tween-20. After taping, subsequent operations should be performed swiftly to keep the plate from drying out for too long.

21. Block the plates with 200 μL of 1 × ELISA/ELISPOT buffer and incubate for at least 1 h on a shaker at 25°C–30°C.

***Alternative:*** 1 × ELISA/ELISPOT buffer can be substituted with 1 × PBS containing 1% BSA or heat-inactivated FBS.

22. Prepare the standards and dilute the samples.
  a. Reconstitute and dilute the standards in serial dilutions.
  b. Dilute the samples with 1 × ELISA/ELISPOT buffer or PBS in an appropriate dilution fold.
23. Repeat Step 20 and then incubate 100 μL of diluted samples or standards in individual wells of the plates for at least 2 h on a shaker at 25°C–30°C.

***Note:*** The dilution fold and incubation time for different samples can be optimized. For detecting IFN-γ, over 16 h incubation of the samples at 4°C is recommended.

24. Repeat Step 20 and add 100 μL of diluted detection antibody to each well, then incubate on a shaker for 1 h at 25°C–30°C.
25. Repeat Step 20 and add 100 μL of diluted avidin-HRP or streptavidin-HRP (horseradish peroxidase) into each well, then incubate in the dark for 30 min at 25°C–30°C.
26. Wash the wells with 200 μL of washing buffer 5 times and tap the plates on absorbent paper towels to remove any residual buffer.
27. Add 100 μL of HRP substrate (TMB; 3,3,5,5-tetramethylbenzidine) and incubate in the dark for 10–20 min.

**CRITICAL:** The incubation of both TMB and HRP should occur in the dark. TMB reaction is monitored in real-time and quenched at appropriate time based on the color intensity of the samples.

28. Add 50 μL of stop solution into the wells to terminate the reaction.

***Note:*** The stop solution is composed of 2 M H_2_SO_4_ or 2 M HCl and stored at 25°C–30°C.

**Figure 4:**
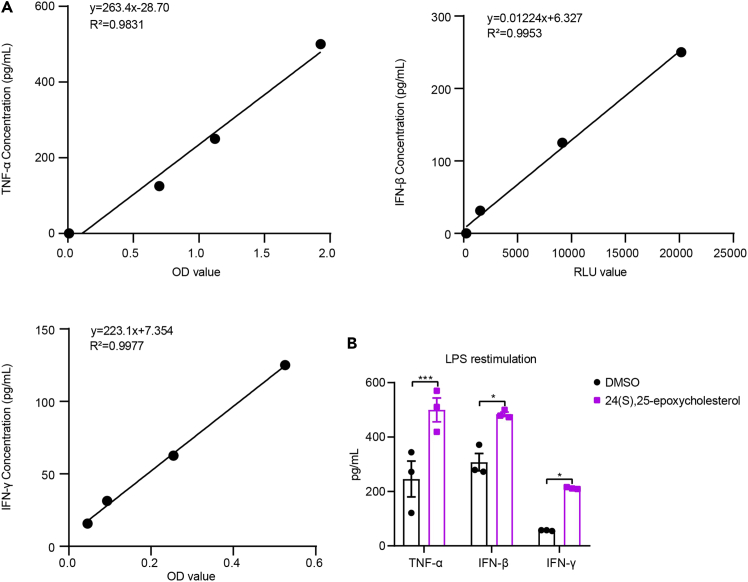
ELISA assay measuring TNF-α, IFN-β, and IFN-γ in the supernatants from 24(S),25-EC-trained BMDMs (A) The standard curve of TNF-α, IFN-β and IFN-γ standards. (B) The supernatant levels of TNF-α, IFN-β and IFN-γ in trained BMDMs after LPS restimulation. Mean ± SEM with three independent experiments, by two-way ANOVA test followed by Sidak’s multiple comparisons test. ∗p < 0.05, ∗∗∗p < 0.001.

29. Measure the absorbance at 450 nm using a microplate reader and set the absorbance at 570 nm as reference.
30. Calculate the concentration of the samples in accordance with the standards (Figure 4).

**CRITICAL:** The dilution fold should be optimized to ensure that the absorbance values of the samples fall within the range of the standards.

### ATAC-seq

**Timing: 30 min (for step 31)**

**Timing: 30 min (for step 32)**

**Timing: 50 min (for step 33)**

**Timing: 30 min (for step 34)**

**Timing: 60 min (for step 35)**

**Timing: 30 min (for step 36)**

The detection of cytokines can reflect the enhanced secondary inflammatory response after training,^8^ but to define the innate immune memory after initial training, the epigenetic changes such as chromatin openness need to tested.^9^ For this purpose, ATAC-seq is performed to investigate chromatin accessibility and to reflect the induction of immune memory. Thus, we extract the nuclei from 24(S),25-EC-trained BMDMs on the last day of the resting period to construct an ATAC-seq library.

31. Collect live BMDMs.
  a. Wash the cells with warm 1 × PBS once, then incubate the cells with Trypsin-EDTA (0.25%) for 1 min at 37°C, and wash the cells again with 1 × PBS twice.
  b. Digest BMDMs with Trypsin-EDTA (0.25%) for 5 min at 37°C.
  c. Stop the digestion with BMDM-CM and collect the cells through centrifugation at 150 × g for 3 min at 25°C.
  d. Count the cells and adjust the cell concentration to 1 × 10^5^ cells in 100 μL of 1 × PBS.
    **CRITICAL:** The cells prepared for the assay must contain over 95% live cells. This is crucial because the nucleosomes in dead cells dissociate, which can potentially skew the actual state of chromatin accessibility and reduce the signal-to-noise ratio (S/N). To exclude dead cells, pre-incubate the cells with Trypsin-EDTA (0.25%) for a short duration can remove the low-adherent dead cells, then centrifuge at a low speed to retain only the live cells. Lastly, we stained the cells with trypan blue^10^ to count and assess the ratio of live cells (above 95%) using the TC20 Automated Cell Counter (Bio-Rad) according to the manufacturer’s instruction.
  e. Centrifuge the cells at 500 × g for 5 min at 25°C, resuspend with 100 μL of 1 × PBS, and centrifuge again at 500 × g for 5 min.
  f. Remove the supernatants carefully and place the semi-dry cells on ice, ready for use.
32. Isolate the nuclei from live BMDMs.
  a. Prepare the ATAC lysis buffer and ATAC washing buffer, and place them on ice for use.
  b. Add 45 μL of pre-cooled ATAC lysis buffer to the tubes and gently pipette the cells up and down for 10 times. Resuspend and lyse the cells on ice for 5 min.
  c. Gently pipette the lysed cells with 950 μL of ATAC washing buffer up and down for 5 times.
  d. Centrifuge the cells at 500 × *g* for 5 min at 4°C.
  e. Carefully place the tube on ice and open the lids to remove the supernatant.

***Note:*** To prevent the loss of nuclei, initially use a 1 mL pipette to leave about 50 μL supernatant. Subsequently, use a 20 μL pipette to remove the remaining supernatant, allowing up to 5 μL supernatant uncollected.

33. Chromatin tagmentation.
  a. Prepare the ATAC tagmentation buffer and place it on ice for use.
  b. Resuspend the nuclei with 40 μL of chromatin tagmentation buffer by pipetting up and down for 10 times, and then transfer the suspension into a new 200 μL PCR tube.
  c. Incubation the tube at 37°C for 30 min in a PCR Amplifier.
  d. Add 5 μL of stop buffer into the PCR tube, vortex thoroughly and incubation again at 55°C for 10 min after a brief centrifugation.

***Note:*** The stop buffer is composed of 5% SDS, and the samples post-reaction can be subjected to purification or stored at −20°C for up to 1 week.

34. Purification of the tagmentated chromatins.
  a. Pre-warm the Tagment DNA Extract Beads in 25°C for at least 15 min.
  b. Vortex the beads thoroughly for 30 s and add 2 volume beads (i.e., 90 μL) into the PCR tube. Mix the beads by pipetting up and down, and incubate at 25°C for 5 min.
  c. Briefly centrifuge the tube and place it in a magnetic hopper grate at 25°C–30°C for 5 min.
  d. Carefully remove the liquid and wash the beads with 200 μL of fresh 80% ethanol twice (1 min for each washing).
    **CRITICAL:** Always keep the tube in the magnetic hopper grate during washing and avoid the beads from being flushed directly.
    ***Alternative:*** If a 1.5 mL tube is used for purification, 1 mL of 80% ethanol is employed for washing each time.
  e. Thoroughly remove the 80% ethanol and dry the beads in the magnetic hopper grate for about 4–6 min.
    ***Note:*** The drying time for the beads varies depending on the environmental temperature, so check the beads in real-time. If the edges of beads turn yellow and the surface become dull, the beads can be considered dry.
  f. Take the tube away from the magnetic hopper grate and add 22 μL of elution buffer to resuspend the beads. Incubation at 25°C for 3 min.
    ***Note:*** If the beads naturally settle to the bottom, use the pipette to resuspend again and incubate for extra 2 min.
  g. Briefly centrifuge the tube and place the tube in the magnetic hopper grate again for 2 min.
  h. Transfer the supernatants (about 21 μL) to a new tube. The purified DNAs can be subjected to amplification or stored at −20°C for up to 1 month.
35. Library amplification.
  a. Prepare the Library amplification mix in a new 200 μL PCR tube.

**Table:** 

Reagent	Amount
Purified DNA	16 μL
i5 index	2.5 μL
i7 index	2.5 μL
2 × HiFi AmpliMix	25 μL
Sterilized ddH_2_O	4 μL
Total	50 μL
  b. Vortex the mix and subject it to a PCR Amplifier after a brief centrifugation and subsequent amplification procedure.

**Table:** 

Steps	Temperature (°C)	Time (s)	Cycles
Strand Displacement	72	180	1
Initial Denaturation	98	30	1
Denaturation	98	15	13
Annealing	63	8	
Extension	72	3	
Final extension	72	120	1
Hold	10	∞	1

**Figure 5:**
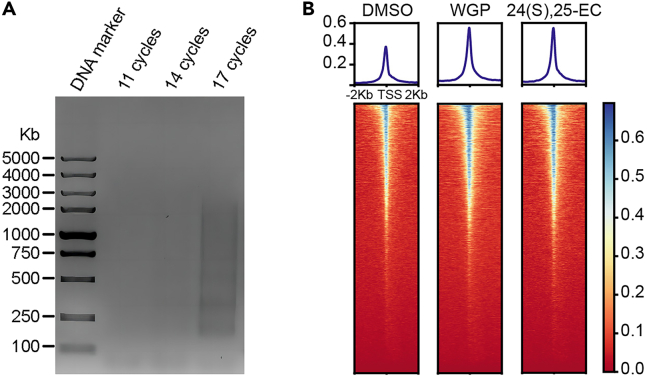
ATAC-seq assay measuring the chromatin accessibility of 24(S),25-EC-trained BMDMs (A) AGE of amplified DNA libraries with different amplifying cycles. (B) Density plots and heatmaps of all peaks depict the global chromatin accessibility around TSS regions in each group. Kb stands for kilobase and TSS stands for transcription start site. WGP serves as a positive control for inducing innate immune memory with increased chromatin accessibility.

    **CRITICAL:** The amplification cycles vary depending on cell type and cell number. To explore the appropriate cycles, 1 μL purified DNA is used to amplify with three gradient cycles (n) and followed by agarose gel electrophoresis (AGE). The cycles (n-4) were determined according to results of AGE, as the detectable DNAs are sufficient for sequencing (Figure 5). Since there may be single-stranded gaps between the adapters and the inserted fragments, a strand displacement reaction at 72°C for 3 min is required to fill these gaps.
36. Purification of the library.

The procedures for purifying library are similar to Step 34 except for that the DNA Clean Beads are used and the volume is 1.3 fold. The purified library is ready for quality control (QC), sequencing, and analysis (Figure 5).

***Alternative:*** The binding of the beads can be divided into two steps to enhance the purity of library. First, bind the PCR products with 0.55 volume of beads, then transfer the supernatants to a new tube containing 0.75 volume of fresh beads. After undergoing secondary binding, the beads are subjected to washing with 80% ethanol.

## Expected outcomes

This protocol aims to establish a method for measuring endogenous metabolites, especially the lipid metabolites, and to examine their impact on inducing innate immune memory. Therefore, the protocol would measure the intracellular level of 24(S),25-EC in mouse macrophages (as shown in Figure 2). Besides, the protocol would also indicate that 24(S),25-EC is cell-permeable (as shown in Figure 3). Moreover, the protocol would show that 24(S),25-EC induces innate immune memory in treated BMDMs, exhibiting generally higher chromatin accessibility (refer to Figure 5B), and displays trained immunity features in trained BMDMs, evidenced by enhanced TNF and IFNs production upon restimulation with LPS (refer to Figure 4).

## Quantification and statistical analysis

The fluorescence data is analyzed using FlowJo software version X10.0.7r2 (Tree Star), and the mass spectrometry data is processed with Agilent MassHunter Quantitative Analysis software (Agilent). The GraphPad Prism version 8.0.1 (GraphPad Software, San Diego, California, USA) is used for further data analysis. Results are presented as the mean ± SEM. Statistical analysis is performed using an unpaired t-test, one-way or two-way ANOVA, followed by Tukey’s multiple comparisons test, as appropriate. All statistical significance and comparisons are detailed in the figure legends.

## Limitations

The *in vitro* model for inducing innate immune memory may not sufficiently reflect the complexity of *in vivo* physiological conditions. This protocol is adapted for mouse BMDMs and needs further optimization for other types of cells or species. The addition of 24(S),25-EC can induce innate immune memory in BMDMs; however, it remains unknown whether 24(S),25-EC secreted by cells can enter and affect BMDMs. Although our previous literature has indicated that 24(S),25-EC induces trained immunity by activating endogenous receptors,^1^ this protocol could not determine whether surface receptors were also necessary, similar to mevalonate^11^ and oxidized low-density lipoprotein (oxLDL).^12^

## Troubleshooting

### Problem 1

The quantity and cell status of differentiated BMDMs are not adequate for subsequent assays.

### Potential solution

It may be due to insufficient levels of the M-CSF for macrophage differentiation; thus it is suggested to increase the proportion of L929 conditional medium or the concentration of M-CSF. Additionally, the ethanol must be thoroughly washed off.^13^

### Problem 2

There are very large variations in the concentration of 24(S),25-EC among the samples in the group.

### Potential solution

To ensure consistency, the cells in replicates should initially be kept in equal amounts. Furthermore, the method of scraping cells should be kept as similar as possible. Additionally, the reagents used for extracting metabolites must be of LC-MS grade to prevent any unnecessary or unspecific contamination.

### Problem 3

The level of cytokines following secondary stimulation in trained BMDMs does not increase compared to that of untreated BMDMs.

### Potential solution

The issue may stem from two potential reasons. One is that 24(S),25-EC is inactive, and the other is that the cells may be contaminated prior to the first stimulation. Therefore, 24(S),25-EC must be divided into aliquots and repeated freeze-thaw cycles should be avoided. Additionally, it is essential to ensure that the mice used are healthy, and that the cells are free from mycoplasma or fungal contamination during the operation.

### Problem 4

The concentration of the library is too low for sequencing.

### Potential solution

The low concentration of the library is primarily due to the loss of nuclei. When extracting nuclei, the pipette should be used to avoid contact with the cells during the removal of supernatants and should be handled with care. Besides, increasing the original cell numbers will improve the concentration of the library. Furthermore, always optimize the amplification cycles to ensure the yield of the library.

### Problem 5

Band dispersion in the testing agarose gel is irregular when amplifying the library.

### Potential solution

It may be attributed to two possible reasons. One is the low activity of the cells, and the other is the contamination of mycoplasma. The DNA of the dead cells or mycoplasma is more exposed than that in clean and live cells, so the chromatins are randomly digested by Transposase without the protection of nucleosomes and become more dispersed. To address this, the cells should be kept clean and dead cells should be removed as thoroughly as possible before nuclei extraction.

## Resource availability

### Lead contact

Further information and requests for resources and reagents should be directed to the lead contact, Jinyun Liu (hy0224009@muhn.edu.cn).

### Technical contact

Details and questions for this protocol should be directed to and answered by Yongxiang Liu (liu_yongxiang@gzlab.ac.cn).

### Materials availability

This protocol did not produce any new materials.

### Data and code availability

This protocol did not generate any new code, and all data were included in the study.

## Acknowledgments

This work was supported by grants from the 10.13039/100014717National Natural Science Foundation of China (32370923 and 32400716) and Young Scientists Program of Guangzhou National Laboratory (QNPG24-03). The graphical abstract was created from BioGDP.com with partial modifications.

## Author contributions

Protocol design, Y.L., J.L., and X.X.; performing assays, Y.L., H.J., and B.H.; writing, Y.L.; revising, Y.L., X.X., and J.L.

## Declaration of interests

The authors declare no competing interests.
